# Caring for home‐dwelling parents with dementia: A qualitative study of adult‐child caregivers' motivation

**DOI:** 10.1002/nop2.587

**Published:** 2020-08-07

**Authors:** Heidi Dombestein, Anne Norheim, Karina Aase

**Affiliations:** ^1^ Centre for Resilience in Healthcare Faculty of Health Sciences University of Stavanger Stavanger Norway; ^2^ Department of Caring and Ethics Faculty of Health Sciences University of Stavanger Stavanger Norway

**Keywords:** adult‐child caregivers, dementia, home‐dwelling, motivation, qualitative method, self‐determination theory

## Abstract

**Aim:**

To explore adult children's motivation in caregiving for their home‐dwelling parents with dementia.

**Design:**

Qualitative design with a phenomenological approach.

**Methods:**

Semi‐structured individual interviews with 21 adult sons and daughters who were caregivers for a parent with dementia. Data were analysed using systematic text condensation.

**Results:**

Inspired by self‐determination theory, three categories were identified in the empirical data representing the main motivational drivers for adult‐child caregivers: *relatedness* (to the parent with dementia, the parent's spouse, other persons), *competence* (in handling dementia, in the parent's need) and *autonomy* (freedom of choice, innate values and tasks). Caregivers report relatedness as their key motivational driver.

These results imply that nurses and other health professionals should value the importance of relatedness when interacting with dementia caregivers and establish belonging support structures. Further research should generate more knowledge of the positive motivational drivers, including interventions to improve relatedness, competence and autonomy.

## 1 INTRODUCTION

Dementia is recognized as a public health priority because an estimated 50 million people are living with dementia worldwide (WHO, 2019). Most of these persons experience that dementia leads to increased impairments affecting memory, personality, meaningful activities, social contacts and self‐care (Bjørkløf et al., 2019). Dementia symptoms can disrupt collaboration between patients and healthcare professionals. For this reason, focus often shifts to informal caregivers and relatives who become important resources for both the person with dementia (PWD) and the professionals (Garcia‐Ptacek, Dahlrup, Edlund, Wijk, & Eriksdotter, 2019). Therefore, it is necessary for nurses and other healthcare professionals to be aware of caregivers' situation to identify potential challenges and initiate support (Chiao, Wu, & Hsiao, 2015; Koren, Laidsaar‐Powell, Tilden, Latt, & Butow, 2018). Informal caregivers of home‐dwelling persons with dementia often experience stress and reduced quality of life because of their caregiver role (van der Lee, Bakker, Duivenvoorden, & Dröes, 2014; Pearlin, Mullan, Semple, & Skaff, 1990). The perceived caregiver burden, caring approach and coping strategies depend on the type of dementia (Svendsboe et al., 2018), caregivers' resources (Chen & Bailey, 2018; Wennerberg, Eriksson, Danielson, & Lundgren, 2016) family relations (Bjørge, Kvaal, Småstuen, & Ulstein, 2017) and received support (Lee, Puga, Pickering, Masoud, & White, 2019). Most research in the caregiver field has examined family caregivers as a homogenous group, without differentiating spouses from adult children of a PWD (Tatangelo, McCabe, Macleod, & You, 2018). Adult children are likely to juggle caregiving and other roles such as work and responsibilities to their own families; spouse caregivers are more likely to undertake a full‐time caregiving role (Conde‐Sala, Garre‐Olmo, Turró‐Garriga, Vilalta‐Franch, & López‐Pousa, 2010). This study therefore contributes to a more differentiated knowledge of caregiving regarding adult children to PWD.

## 2 BACKGROUND

So far, research has focused on the challenges of being a caregiver to a PWD. There is a need to draw attention to other elements of caregiving (Lloyd, Patterson, & Muers, 2016) such as satisfaction, autonomy and expertise (Yu, Cheng, & Wang, 2018). Motivation for caregiving in the dementia context is crucial in informal carers’ experience of their role (Quinn, Clare, & Woods, 2015) as it energizes behaviour, initiate, generate and increases task engagement and direct actions. Motivation is thus understood as the energy in people that drives their actions or non‐actions. Motivational support increases caregivers' sense of well‐being, psychological growth and resilience (Weinstein & DeHaan, 2014). In a review article, Greenwood and Smith (2019) found the motives of family caregivers to persons with dementia, for example reciprocity, commitment, love, duty, loyalty, obligations and responsibility, to be identical with their reasons to sustain as caregivers. Still, the motivations for taking on and staying in the caregiver role remain an area not fully understood (Greenwood & Smith, 2019; Quinn, Clare, & Woods, 2010). Thus, studying caregiving using a theoretical motivation framework is recommended as it can further help identify and categorize motivational aspects (Quinn et al., 2010).

In this study, caregivers' motivation is addressed using self‐determination theory (SDT) (Ryan & Deci, 2000, 2017) whose premise is the three psychological drivers of motivation: the need for autonomy, competence and relatedness. Their satisfaction would be essential for individual psychological growth, subjective well‐being and optimal human functioning, while thwarting those needs can lead to amotivation (Ryan & Deci, 2000, 2017; Weinstein & Ryan, 2010; Williams et al., 2014). When the need for *autonomy* is satisfied, one experiences a sense of volition and the sense that one's actions are endorsed by oneself, conferring a feeling of ownership over actions. The need for *competence* relates to mastery and perceptions of performing tasks with confidence, effectiveness and being capable of achieving desired outcomes. The need for *relatedness* is a feeling of mutual belonging, genuine connection with others and experiencing giving support to and being supported by others (Ryan & Deci, 2000, 2017).

Self‐determination theory has been applied to several healthcare contexts (Ng et al., 2012; Ntoumanis et al., 2020) such as caregivers' motivation for persons with cancer (Kim, Carver, & Cannady, 2015; Ng, Griva, Lim, Tan, & Mahendran, 2016) and chronic pain (Kindt, Vansteenkiste, Cano, & Goubert, 2017) as well as to caregivers caring for relatives with different long‐term illnesses (Dombestein, Norheim, & Lunde Husebø, 2019). To our knowledge, SDT has not yet been applied to adult children caring for home‐dwelling parents with dementia. There is a lack of qualitative studies expanding the SDT framework and understanding the phenomenon of caregivers’ motivation, (Ng et al., 2016) also in a dementia context (Pierce, Lydon, & Yang, 2001). Therefore, this qualitative study explores adult children's motivation in caregiving for their home‐dwelling parents with dementia. This aim will be addressed through the following research question:

*How can adult children's motivational drivers for caregiving be described using self‐determination theory?*

## 3 METHODS

### 3.1 Context

In Norway, about 80,000 persons live with dementia. Approximately the same number are primary caregivers for these persons, while about 270,000 are secondary caregivers (Norwegian Ministry of Health & Care Services, 2015). As in other Nordic countries, Norway's public healthcare system is constructed for taking care of PWD with supplements from informal caregivers. Specialist care is provided by regional health services and consists of hospitals and specialized units such as memory clinics and geriatric hospital wards. More than 300 Norwegian municipalities are responsible for primary care like general practitioners, home care, day care centres and nursing homes (Norwegian Ministry of Health & Care Services, 2018). In Norway, adult children are not legally required to assume care of their parents, but many do (Bøckmann & Kjellevold, 2015). The care provided by adult children to PWD includes for example transportation, assistance with practical tasks, support for personal care and medical treatment, but mostly emotional support including spending time talking with the parent, visiting or calling to ensure that the parent is safe. In the home‐dwelling period, home care services are common, often starting small and increasing as the dementia progress. PWD usually live at home as long as justifiable possible but moving the parent into a nursing home is often normal at an advanced stage of the illness (Norwegian Directorate of Health, 2018). The participants in this study had home‐dwelling parents with dementia in different parts of a large municipality in Western Norway containing urban areas and rural districts.

### 3.2 Study design

This research study adopted a qualitative design (Malterud, 2001; Polit & Beck, 2018) with a phenomenological approach (Creswell & Creswell, 2018) to explore the lived experiences with motivation in the caregiver role as described by the participants. The method for data collection was individual face‐to‐face interviews (Polit & Beck, 2018). This was chosen out of consideration for the participants who would be sharing personal information on a potentially sensitive topic. Data were analysed by systematic text condensation (Malterud, 2012). The COREQ, 32‐item checklist is used in reporting the study (Tong, Craig, & Sainsbury, 2007).

### 3.3 Participant selection and recruitment

Purposive sampling (Polit & Beck, 2018) was used and involved selecting participants who shared particular characteristics and had the potential to provide rich, relevant and diverse data pertinent to the research question. To be included in the study, the participants had to be over 18 years old and registered as a primary or secondary caregiver as their parent diagnosed with dementia was receiving healthcare services in the municipality. In addition, the parent should have moved to a nursing home for between 2 and 12 months prior to the interview. The reason for conducting retrospective interviews was that the last home‐dwelling period—waiting for a place in the nursing home—could be especially stressful for both patients and their caregivers. Caregivers might find it difficult to express the positive elements of the caregiver role. Thus, by interviewing the caregivers at least 2 months or more after the parent has moved to a nursing home, the caregiver will have had the time to create some distance from the parents’ home‐dwelling period and be better able to reflect on the situation, add meaning to this experience and articulate it. The upper limit (12 months) was set so the participants could still remember their experiences with the caregiver role. Adult daughters and sons who met the inclusion criteria were identified and recruited by a project nurse working as a coordinator in the community. She was instructed to recruit both male and female caregivers. She telephoned 31 people. Five declined to participate, claiming that they did not have the time, energy or capacity to do so. Author HD called the participants who had agreed to be contacted by the researchers and of those five declined to participate for the same reasons. In sum, ten people who were asked to participate declined. 21 persons consented to be interviewed and none withdrew from the study.

### 3.4 Sample

The participants were 12 daughters and nine sons. Biological, adoptive‐ and foster children were given equal status. At the time of interest when the parent with dementia was still living at home, none of the participants had been sharing a household with the care recipient. 12 of the participants had parents living alone, and in nine cases, the parent with dementia had lived with her or his spouse. All caregivers were holding paid jobs in addition to being informal caregivers. Table 1 contains information on the participants.

**TABLE 1:** Characteristics of participants, *N* = 21 (%)

Characteristics	*N* = 21
Gender, *N* (%)	
Female	12 (57)
Male	9 (43)
Age group, years, *N* (%)	
40–49	5 (24)
50–59	11 (52)
60–69	5 (24)
Education, *N* (%)	
Public school	2 (10)
High school	5 (24)
University	14 (66)
Job status, *N* (%)	
Full‐time	19 (90)
Part‐time	2 (10)
Retired	0 (0)
Parents' household status, *N* (%)	
Mothers living alone	7 (33)
Mothers living with spouse	5 (24)
Fathers living alone	5 (24)
Fathers living with spouse	4 (19)

Note

Table 1 shows the variation in caregivers' age, gender, education, job status and the parents’ household status.

### 3.5 Interview setting

The interviews took place at times and places that were convenient for the participants, such as their workplace, their home or a meeting room at the university. A few of the interviews started with the participants seeming a bit nervous, but after a while, a trusting atmosphere was established where the participants were able to share their experiences and express their opinions. In each interview session, only the participant and the interviewer were present and the participants seemed willing to speak openly.

### 3.6 Data collection

A semi‐structured interview guide (Polit & Beck, 2018) with open‐ended questions was informed by previous research, the self‐determination theory and through discussions in the research group. The interview guide was tested on a daughter who met the inclusion criteria and she contributed to the interview guide and the interview itself. For example, she found it hard to answer the vague question of *why* she had put so much effort in helping her father. She recommended breaking the question into smaller themes to encouraging participants to start talking about their motivation. She requested a more precise question about her relationship to her father before and after the dementia diagnosis; her knowledge of dementia and of her father's symptoms; and the importance of being able to decide for herself what she should and should not do as a caregiver. Her feedback led to a more detailed and expanded interview guide [see Appendix S1]. This pilot interview contributed with rich data and was therefore included in the sample.

Data were obtained from individual face‐to‐face interviews (Polit & Beck, 2018) conducted by HD in 2017. HD is an experienced nurse trained in interviewing and consulting elderly patients and their caregivers. All participants were informed that she was a PhD student writing her PhD thesis on caregivers. The interviewer was unknown to all the participants except for the pilot interview; that participant was an acquaintance. The same interview guide was used in all interviews except for the pilot interview. All participants were interviewed once and each interview lasted from 56 min to 1 hr and 47 min; the median length was 1 hr and 11 min. The data material was digitally audio‐recorded and later transcribed, mainly by HD. A professional transcriptionist was hired to transcribe parts of the last seven interviews. The transcripts were not returned to participants for comments as their intuitive experiences were considered essential for the research question. Permitting elaboration and corrections could have resulted in a data set that did not represent spontaneous answers to the interview questions.

The recruitment process lasted for several months. The participants were interviewed until no new relevant knowledge was generated (i.e. after 21 interviews). The research group discussed the point of data saturation (Polit & Beck, 2018). A bias in retrospective interviews might be that some participants easily could switch from the past to the present time and talk about the parent in the nursing home. Therefore, there was a risk of obtaining irrelevant information. This was handled in the interview situation by asking the participants to recall examples from the home‐dwelling period. Data on caregiving after the parent had moved to a nursing home were excluded from the analysis because it was not relevant to the research question.

### 3.7 Analysis

Systematic text condensation, a four‐step method for thematic analysis of qualitative data (Malterud, 2012, 2017), was adopted because we wanted to explore the sustained motivation across caregivers. A single designated participant might have illustrated a typical case but not demonstrated variations in caregivers' motivation. Analysing the data consisted of the following steps: (a) reading all of the data material to obtain an overall impression, identifying preliminary themes; (b) identifying meaning units representing different aspects of themes and describing codes and code groups; (c) condensing the contents into subgroups; and (d) summarizing the content of each subgroup.

The three first phases of analysis had an open approach. Once the themes, code groups and subgroups were identified and described, the SDT framework was an aid when categorizing subgroups and finding category headings in the fourth step. In this way, the analysis was data‐driven, but the SDT helped to weave the grouped data into meaningful categories. This deductive approach of placing subgroups together in the fourth step may risk the exclusion of relevant data (Overgaard & Bovin, 2014). This issue has been addressed by collecting, summarizing and reporting findings not categorized according to the SDT. Two authors (HD, AN) read all the transcribed interviews and the third author (KA) read one‐third of them. The three authors independently listed the emerging themes and through discussions agreed on preliminary themes. HD identified meaning units and quotes reflecting each theme and developed descriptions of code groups. The descriptions reflecting the code groups were discussed among all authors before reorganizing, renaming and eliminating code groups. Subsequently, consensus on three categories was achieved after four analysis workshops where all authors participated. The data material was organized with NVivo 12 (QSR International, 2019) that was used to systematize 402 pages of transcribed data material.

HD is a registered nurse with a MHSc in ageing and dementia, working with people with dementia and their caregivers for several years. She assumed that being a caregiver is stressful and it might be difficult to see the positive sides of caregiving. Author AN is a registered nurse and professor of nursing science; and author KA is an engineer and professor of quality and patient safety. The authors' range of backgrounds led to fruitful discussions, bringing new perspectives and preventing the first author's assumptions from leading to bias in the analysis process.

### 3.8 Ethics

The PhD thesis of which this study forms a part has been approved by the Norwegian Centre for Research Data (NSD), reference number 48,276. The study has been conducted according to the recommendations of the Helsinki Declaration (World Medical Association, 2013). The project nurse gave oral information to possible participants. Those who agreed to receive more information got a letter with details about the study stating that participation was voluntary, including a description of how confidentiality and anonymity were ensured. The participants signed and returned a written consent form to the research team. Identical information was verbally repeated at the start of each interview, and participants were also reminded that they could stop the interview or withdraw from the study at any time without stating any reason.

## 4 RESULTS

All 21 of the adult‐child caregivers willingly told their caregiver story, reflecting on the reasons for helping their mother or father. In the following, we describe the findings of our analysis according to the three main categories of *competence*, *autonomy* and *relatedness* with the associated subgroups (Table 1, 2).

**TABLE 2:** Categories, subgroups and descriptions of caregivers' motivational drivers

Category	Subgroups	Descriptions
Competence	The parent with dementia's needs	Thorough knowledge of the parent to identify what he/she needs. Different levels of coping and capacity in performing tasks for the mother/father and in meeting their needs.
	Handling dementia	Knowledge of the dementia illness and related professional help affects caregivers’ feelings of mastery or helplessness in their role.
Autonomy	Freedom of choice	The ability to choose when and how to help the parent.
	Innate values and tasks	The naturalness of performing tasks to help the parent without thinking about why they do so, combined with a nuanced feeling of a sense of duty.
Relatedness	The parent with dementia	The relationship with the parent before and after the dementia diagnosis and how well the adult child thrives with the parent.
	The parent's spouse	The close relationship with the healthy parent, mutual belonging and support to the parent with dementia's spouse having the primary burden of daily caregiving.
	Other persons	Collaboration and relationships with other people such as siblings, friends, colleagues and healthcare professionals. Being listened to, treated with respect and receiving support.

### 4.1 Competence

Caregivers described their competence as the capability to master their daily life with the PWD. The competence of the adult children allowed them to experience different levels of control and predictability in their role as caregivers, thus affecting their motivation to remain in the caregiver role.

#### 4.1.1 The parent with dementia's needs

All caregivers experienced their parent gradually losing the ability to perform daily activities. The adult children were therefore in contact with their parents at least once a week and sometimes several times a day, trying assist their parents with activities of everyday life:

It started with her not being able to pay her bills because she couldn't handle the internet anymore and she needed help to pay bills and sort out her finances. Then she needed help running errands because she lost her driver's licence and she couldn't get around as she used to. Then it piled up with different needs like help with taking her tablets, grocery shopping, preparing meals and remembering to eat them. She needed help cleaning her apartment, doing her laundry and eventually she didn't know how the shower worked so she needed help with her personal hygiene and so on and so on…. (Daughter, participant G)

Knowing what the parent needed was useful for the caregiver, but not being able to meet those needs was frustrating and thwarted their motivation. When caregivers succeeded in meeting their parent's needs, they felt competent, satisfied and pleased with their capacity to do so: *“I could see that my effort was helping her and that is motivating in itself. Then helping her was not an energy loss, but gave me good energy”* (Daughter, participant C).

The mastery of meeting the PWD's daily needs for support was an essential driver for motivation among adult children: *“What's motivated me as a caregiver was that my mother should have the best possible life while staying at home”* (Son participant M). A daughter illustrates her expertise on her mother's needs: “*Making her feel as good as possible was driving me to meet her needs. When I knew her so well, I could see what she needed and I could see when she was happy and feeling ok.”* (Participant U).

#### 4.1.2 Handling dementia

Several of the adult children initially described a feeling of incompetence and helplessness when they did not know the reason for the decline in the parent's cognitive function and behaviour. At the same time, they were struggling to get access to professional help. Therefore, it was a relief to receive the diagnosis and obtain knowledge on how to handle the symptoms. To have competence in dementia and feel a sense of mastery and effectiveness when helping the parent was an important source for their motivation. A daughter was frustrated and exhausted by trying to get her father to wear clean clothes, but after the diagnosis, she understood how to handle this behaviour:

If I feel that this is working, what I'm doing is making my father better in one way or another, then I have a feeling of mastery and it gives me something. When it doesn't work, you somehow lose that motivation, you go on the same track over and over again and it gets worse and worse actually. So I think maybe that is where mastering is important and that is part of my motivation. (Participant R)

Some caregivers felt a lack of competence, confused and lost in the health system trying to obtain professional assistance for their parent sometimes leading to a sense of amotivation:

At first I didn't know where to start. I spent a lot of time and effort trying to find out where and how to get help for my mom… It felt like a waste of energy to struggle against the system. I wish I had known five years ago what I know now and then I would have avoided spending so much time and effort trying to figure things out. (Daughter, participant V)

Knowing how the healthcare system is organized and whom to contact in different situations was an advantage in caregivers gaining a sense of perceiving control, predictability and competence. Competence in dementia and the healthcare system was obtained in different ways using various sources. Only a few caregivers had attended dementia information meetings or courses; others had consulted the internet or booklets on dementia. Common among caregivers was the helpfulness of information and advice from friends or colleagues whose own parents had had dementia:

I often went hiking with a friend of mine. Her mother recently died of dementia… We frequently talked about what I could expect at different phases of the illness, how to collaborate with the health care professionals and what I could say and do to handle my father's sometimes challenging behaviour. It was good to talk to my friend and she supported me for years. (Daughter, participant J)

### 4.2 Autonomy

The adult children expressed the importance of the ability to choose when and how to help the parent as essential for their motivation in the caregiver role. At the same time, values and nuances of sense of duty were innate when describing tasks as natural.

#### 4.2.1 Freedom of choice

All caregivers agreed on the importance of deciding for themselves with which tasks to help their parents. Not taking orders but voluntarily performing tasks was a driver: *“If someone is squeezing you like a lemon on what you have to do, I would probably just shake it off and say, ‘No, I want to decide for myself’. It was my own will or my own motive to help my parents, which I really have no other answer to”* (Son, participant E). Another son stated: *“I feel that what I did, I did it of my own free will, I could have said no”* (Participant F).

It was also important for caregivers to have the opportunity to set boundaries for not accepting to perform specific tasks: *“To help my mother was perfectly fine up to a certain point. Therefore, I couldn't go into the shower with her. It got too close”* (Daughter, participant G).

Several caregivers mentioned the uncertainty around the point where it was time to level up and accept more help from professionals in the PWD's home. Some daughters had felt guilty but were content that they had decided to accept help:

I think since we got the offer, I chose to use it to be able to be the daughter who is not completely exhausted. So I would rather be the one visiting him and socialising with him instead of being a nurse and a home maid. I could probably have done more, but I chose to receive help. I know myself pretty well eventually, having to make my choices, be a little conscious that you need to be yourself as well. (Daughter, participant A)

#### 4.2.2 Innate values and tasks

It was challenging for caregivers to explicitly describe their motivation for taking care of their father or mother with dementia. Everyone described it as a “*natural”* thing to do and *“that is how we do it in our family, so the values are inherited”*. They had never thought of it as an alternative not to involve themselves in caregiving: *“No, that's just the way it should be, she is my mother! So I never thought about that”* (Daughter, participant U).

These descriptions indicate caregiving as part of their innate values and tasks. Eventually, after talking more about the topic several caregivers expressed how they felt it valuable and important to perform tasks and help their parent and how this was done without a feeling of obligation, this feeling was part of their *“backbone”*:

I think motivation comes from what I have learned, experienced or what is right to do, what makes you the person you are. Of course, it's your whole life that has influenced you and the motivation for caring for my mother lies within myself. (Daughter participant T)

None of the caregivers reported that someone outside or inside the family had told them to help their parent, but several had felt the pressure to do so. The desire to take care of their parents came mostly from within themselves but sometimes from a sense of duty:

My father took it as a matter of course that I should provide care for him, he probably did. I never thought that there was any compulsion in a way, but there wasn't anyone else. So in a way, maybe indirectly… It can be an expectation from others and an expectation from yourself, what you expect of yourself. (Daughter, participant J)

Most of the caregivers seemed to have accepted these sentiments and they were not preoccupied with the thought of having acted out of a sense of duty or out of free will. In retrospect, it was more important to be able to look back on the home‐dwelling period with the certainty of knowing they had chosen to do what they could to help their parent: “*It felt like the right thing to do”* (Son, participant P).

### 4.3 Relatedness

The caregivers talked first and foremost about their relations with other people when describing their motivational drivers. Here, gaining positive energy from interacting with the parents and other persons was central to their motivation. A feeling of belonging being a respected part of a team was essential for remaining motivated as a caregiver.

#### 4.3.1 The parent with dementia

All caregivers talked about the relationship with the parent before they developed dementia. None of the adult children in this study indicated that they had previously had a markedly difficult relationship with that parent. Instead, they stated that they were genuinely fond of their parent and this made it easier to help them even with the less pleasant tasks:

If your mother has been fond of you, then you are motivated to return that kindness and when she begins to struggle, you are much more motivated. You've had a good relationship all these years and that makes you contribute to something you don't think is very nice, such as going home to her when she was living at home then, picking up clothes that she had peed in and taking them home and washing them. (Son, participant O)

Some were concerned with the relationship after the dementia diagnosis. The PWD could be discontented and bad‐tempered and then the caregiver sometimes felt like a nagging child not being welcomed in their parent's house:*“…on those days the visit to mom did not give me positive energy”* (Son, participant D). A daughter had similar experiences: *“When I think about how tired I became from helping mom, it may not be how much time I spent, but how much energy I used”* (Participant C).

Several caregivers had not experienced difficulties when visiting and helping their parent. Some said that their parent had never made them feel guilty and were just grateful for the help they received. It was also seen as confirmation of a good relationship when socializing and other ways of helping the parent were perceived as enjoyable. Having a good relationship and enjoying time spent with the parent was an important source of motivation:

We've always had a good relationship and his behaviour was my motivation to visit him. So we had enjoyable times together and it motivated me. But had it been harder to visit him, then I would probably not have had the motivation to see him as much as I did. (Daughter, participant B)

#### 4.3.2 The parent's spouse

Both parents of nine of 21 caregivers were alive and the parent with dementia had lived with his/her spouse. In most cases, these spouses had been in good health and had been the one taking on the major burden of daily caregiving with the support of the adult child. These adult children pointed out the close relationship with the healthy parent as a key reason for their willingness to offer support:

I felt satisfied when I had contributed in a way that was good for my mother or for instance, made her happy, then I was motivated by this. Mother wouldn't have been able to keep him [the participant's father] home unless I had supported her. However, I think even more that I have seen in retrospect with what mom was struggling. She didn't want to worry me either, so it's her way of showing her care for me. So to make sure that I could live my life she took most of the burden until she couldn't do it anymore. Mom probably had no regular sleep for the last 4‐5 months, which meant that she was also completely exhausted, so I was, in the end, afraid that she would somehow end up with a heart attack or similar. (Daughter, participant S)

These caregivers wanted to support the healthy parent. They appreciated gratitude but did not necessarily expect it. Several gave examples of the healthy parent's appreciation of being able to share the responsibility:

Mother was very positive and she is like that by nature. She also wrote a small booklet on 40 pages over the last two years where she talks about her experiences and her thoughts. She writes very positively, so it's her way of saying thank you for the period in which we contributed. (Son, participant K)

#### 4.3.3 Other persons

The responsibility for caregiving was often shared involving not only the adult child (and the PWDs spouses), but also other people in their social network like the caregiver's siblings, other relatives, the caregiver's spouse or grown children. Having respect, understanding and support from these other persons were important:

I had a spouse with an extensive understanding of my situation. He was supportive and never accused me of not being at home and stuff like that. During times when I thought it was mentally difficult, he has been invaluable. I've been the only caregiver for my mother, but I've always had him as support. (Daughter, participant T)

Good relations with other caregivers gave a feeling of belonging, being trusted and meaning something to others. To experience themselves as an essential part of a team caring for the PWD was important to the caregivers. The caregiving also had positive outcomes, like bringing siblings closer:

We are a family with mother, father and four siblings … We have been in a situation where the family has been central and we have been very focused on caring for each other and being friends. There are no conflicts. We have spent a lot of time reflecting on how this has changed us and what has changed. We siblings have actually become even closer. We talk about other things and feelings more than we did before. (Daughter, participant S)

The caregivers rarely mentioned their relations to healthcare professionals. If they did mention these professionals, the relationship was usually negative but with some exceptions. These professionals could have been a family doctor, a community nurse, a professional at the day care centre or a service coordinator. The caregivers expressed being treated with respect, acknowledgement, understanding and support. A son mentioned that even though his father's health services were not always delivered as planned, he was satisfied with the long‐term follow‐up:

We had telephone conversations on demand and she listened to me… She was, as I experienced her, genuinely concerned with trying to find alternatives and things that could help. So, she was very good indeed. (Son, participant E)

### 4.4 The importance of telling the caregiver story

To prevent the exclusion of important finding when using a deductive approach (Malterud, 2012) in our fourth stage of analysis (see 3.7 Analysis), the transcribed material was searched for data that might fall outside the findings of the current analysis. In that respect, we found that caregivers were devoted to telling their caregiver history concentrating on practical issues, psychological stress, lack of support and respite services in their everyday assistance to their parent with dementia and how this also influenced their motivation negatively. Most caregivers had never had someone outside the family take an interest in them as caregivers. Therefore, they said it felt good to speak about what was important to them. In this analysis, these issues were not described in detail as they were outside the scope of the study.

## 5 DISCUSSION

Three categories represent caregivers' main motivational drivers: *relatedness*, *competence* and *autonomy*. Despite the challenges and burden, the adult children in this study expressed positive reasons for becoming and remaining caregivers for their parents with dementia while they were still living at home. Their relatedness to other persons was the key driver for motivation. Relatedness included the importance of being treated with respect, understanding, acknowledgement, being listened to and supported. Other drivers included having competence on dementia and resources to help the parent, often gained through relatedness with others. At the same time, caregivers needed to feel autonomous and to voluntarily perform their caregiver tasks.

Relatedness to the person with dementia is a well‐known factor for how caregivers experience their role (Bjørge et al., 2017; Bjørge, Sæteren, & Ulstein, 2019; Quinn et al., 2015). Relationship quality is directly linked to motivations for providing care and associated with the meaning of caregiving (Greenwood & Smith, 2019; Quinn et al., 2015). Essential in our study was the fact that adult children with two living parents were motivationally driven by the relationship with the healthy parent defining their role as supporting the primary caregiver. Greenwood and Smith (2019) found some similar caring motives described by spousal and adult children like, for example reciprocity, commitment, love, duty, loyalty, obligations and responsibility. On the other hand, previous empirical research has documented different experiences between caring for a parent or a spouse with dementia and between being a primary caregiver or a support for the primary caregiver (Conde‐Sala et al., 2010; Tatangelo, McCabe, Macleod, & You, 2018). This is confirmed in our study from the perspective of adult‐child caregivers.

The adult children in our study reported the importance of relationship quality with other persons (family members, friends, co‐workers, healthcare professionals) where the essence was to be met with respect, understanding and acknowledgement, being listened to and supported. Consistent with self‐determination theory (Ryan & Deci, 2017), this relatedness gave them a feeling of meaning something to others and being an important part of the team caring for the parent with dementia. Thus, satisfying the psychological need for relatedness allowed the caregivers to thrive and become more enthusiastic about caregiving (Pierce et al., 2001).

Our study also confirmed that positive and supportive relationships were important drivers for increasing adult‐child caregivers' competence in assisting parents with dementia. Common was the usefulness of retrieving information and advice from friends or colleagues whose own parents had dementia. This led to the sense of mastery in their daily life with the parent with dementia. Pierce et al. (2001) described how caregivers, who considered themselves as competent, experienced more meaning and enthusiasm in their role. The caregivers in our study sometimes felt incompetent and amotivated when they repeatedly tried to do their best to help their parents, but the situation still became worse. To avoid amotivation, it is necessary for caregivers to understand dementia and have the resources to do so (Chen & Bailey, 2018; Conde‐Sala et al., 2010; van der Lee et al., 2014). According to Williams and colleagues (2014), amotivational behaviour is the belief that there is no clear connection between the individual's performance of an activity and the outcome.

One way of avoiding amotivation is to experience self‐determination and autonomy and caregivers should decide for themselves which tasks to perform (Ng et al., 2016). In our study, self‐determination was expressed by caregivers' ability to decide which tasks they would do to help their parents and when to do them. Feeling obligated to assume in caregiving responsibilities can lead to a heavier subjective burden among adult children (Conde‐Sala et al., 2010; Tatangelo, McCabe, Macleod, & Konis, 2018). Therefore, being autonomous in the caregiver role is important and, in our study, adult children often seemed to have internalized the value of caregiving, performing tasks without talking about the need for autonomy. Looking back on their parents' home‐dwelling period, they expressed that whether they had become caregivers willingly or out of a sense of duty was not important to them. The most valuable motive for them was knowing they had done the right thing by keeping their parent comfortable at home for as long as possible. These findings differ from a main assumption of the SDT framework, where being autonomous and self‐determined are premises for high‐quality motivation (Ng et al., 2012; Ryan & Deci, 2017; Weinstein & Ryan, 2010). In work‐related contexts, satisfying the needs for autonomy, competence and relatedness is valued as equally important (Williams et al., 2014), as is the case for the long‐term caregiver context (Dombestein et al., 2019). In this study, dementia caregivers' needs for competence and autonomy were important, but not as important as relatedness. In other health contexts, use of self‐determination theory has been well‐tested with individual patients as the focus (Ng et al., 2012). Caregiving implies a relationship between the giver and the recipient of care. Reinforcing this, the adult‐child caregiver and the parent with dementia are parts of a community relating and collaborating with family members, healthcare professionals and others.

### 5.1 Methodological considerations

Limitations of the qualitative approach applied in this study should be noted. On the one hand, semi‐structured questions provided in the interview guide may have influenced the final categories, in contrast to allowing the participants to speak freely about their experiences without any prompts. On the other hand, the phenomena motivation is an abstract concept and according to the pilot interview, it was hard to answer open questions like: “What made you help your mother/father with dementia when she/he was living at home?” The questions in the interview guide might also have led the participants to focus mostly on the positive aspects of caregiving, substantiating a possible exclusion of negative aspects and barriers to motivation. There is also a bias in using retrospective interviews as the data collection could obtain irrelevant information or miss vital information. We handled these issues by focusing on specific episodes from the parent with dementia's home‐dwelling period and by excluding information not related to the home‐dwelling period from the analysis.

Another limitation might be that since 10 of 31 potential participants declined for various reasons, we can discuss if our sample, in fact, was a convenience sample. According to Tong et al. (2007), this sample may have failed to capture important perspective from “difficult‐to‐reach” participants. Our results might have been different if these participants had been interviewed and we had the knowledge of their experience in the caregiver role.

The last issue was caused by ethical considerations. We were not allowed to collect data on recipients of care and therefore we had no information on, for example, the type of dementia they had. Adding this information to our study could have strengthened our findings and contributed to the elaboration of possible differences in motivation, for example, in being a caregiver to a parent with Alzheimer as opposed to a parent with frontal temporal dementia. Further research may explore these issues if ethical approval allows it.

## 6 CONCLUSION

In this study, we demonstrated that adult‐child caregivers report relatedness as the key motivational driver for performing their caregiver role for home‐dwelling parents with dementia. The knowledge of motivational drivers presented in this study can inform the work of nurses and other health professionals in dementia care. They should value relatedness when interacting with dementia caregivers and establish belonging support structures such as systematic involvement of the adult children in the parents’ healthcare services or tailored respite care to the parent when needed. From our results, a possible intervention given to adult‐child caregivers could be psychoeducational programmes aimed at increasing their competence on dementia in addition to providing them with customized support and guidance. In our study, we investigated motivation at an individual level, including support and acknowledgement from private networks, colleagues and healthcare professionals. Future research could examine how societal attitudes and other macro‐level factors affect adult children's’ motivation for caregiving. Further research should increase knowledge of the positive motivational drivers for adult‐child caregivers including interventions to improve their relatedness, competence and autonomy. It could also be interesting to study the interconnection of the SDT core aspects in a dementia caregiver context as this has not been done before.

## CONFLICT OF INTEREST

The authors declare that they have no competing interests.

## AUTHORS' CONTRIBUTIONS

Authors HD and AN contributed to the conception and design of the study. HD was responsible for recruitment, data collection, data analysis and drafting of the manuscript. AN and KA participated in four analysis workshops and contributed in the data analysis and interpretation of the data material. All authors critically revised the manuscript and have read and approved the final version of the manuscript.

## ATTACHMENT/SUPPLEMENTARY MATERIAL FOR ONLINE PUBLISHING

Interview guide.

## Supporting information

App S1
