# Supplementary material for: Caring for home‐dwelling parents with dementia: A qualitative study of adult‐child caregivers' motivation
Source: Nurs Open. 2020 Aug 7;7(6):1954–65. doi: 10.1002/nop2.587 (PMC7544851; doi:10.1002/nop2.587)
Supplement: Supplementary file 1 — App S1 [file NOP2-7-1954-s001.docx]

**Appendix S1**

**The interview guide** (Additional file for online publication)

At the beginning of the interview, I will remind the participant that it is the caregiver who is the focus of the interview and not the patient. I will also like to mention that I have not received consent from the parent to obtain information about them. Therefore, third part information should be minimised.

**1. Can you tell me about your everyday life and how it was for you when your mother/father lived at home with dementia?**

Possible follow-up questions:

-What practical tasks and how often did you help your mother/father in everyday life?

-Did you do something social or other things together with your mother/father, can you tell me about it?

-Did your mother/father have other persons than you to help her/him, if so, who?

**2. What made you help your mother/father while she/he lived at home?**

Reformulation if necessary:

-What were your drivers to help your parent with dementia?

Possible follow-up questions:

-Did you experience something positive for yourself by caring for your mother/ father, can you tell about it?

-Did you get any positive feedback and appreciation from other persons for your efforts? If so, how and from whom?

-Did you see your caregiving as meaningful? If so, can you give examples? Did you find that you made a difference to your mother/father when she/he lived at home? If so, in what way?

- Have you ever felt pressured to take care of your mother/father? If so, can you tell about it and from whom you felt pressure?

- Has your relationship with your mother/ father changed after she became ill? How do you think your relationship with your mother/ father has affected your efforts in the caregiver role?

**3. What helped you endure and stay in the role of caregiving during the years when your mother/father lived at home with dementia?**

Possible follow-up questions:

- From where did you get information about dementia? Have you attended a dementia course for caregivers or similar? If so, can you tell me about it?

-If you experienced support from (possible) workplace, your private network or health professionals, can you tell me about it?

-Did you get any kind of relief, e.g. daycare centre or respite services in nursing homes for your mother/father?

-Did you receive any kind of financial compensation for the caregiver work, for example in the form of care pay, leave with pay, pension points or the like?

**4. Think about the challenges you have described. What made you still go on?**

Reformulation if necessary:

- Adult children have no legal duty to take care of old parents in Norway, what made you continue to help your mother/father despite the challenges?

**5. Are there any other needs or associations about being a caregiver that you think is important to mention?**

THANK YOU for participating in this study!
